# Moving chemical sciences together

**DOI:** 10.1093/nsr/nwab005

**Published:** 2021-01-19

**Authors:** Gilberte Chambaud

Chemistry is everywhere and in everything! It is concerned with all the issues that humanity has to face and try to solve, including health, environment and energy. How could we imagine that we can achieve that without strong collaborations stimulating scientists of various cultures! In the present days, we are witnessing the universal and accelerated effort to fight against the coronavirus, working together to find an efficient vaccine as soon as possible. In chemistry also, we realized a long time ago that it is necessary to associate our strength in a long-lasting process advancing stepwise in a collaborative way.

Between France and China, the collaborations in chemistry started more than 40 years ago. Since then and continuously, bilateral activities are growing. In France, one of the major actors of international collaboration is the Centre National Recherche Scientifique (CNRS). The first official bilateral agreement was signed by CNRS with the Chinese Academy of Sciences in 1978, followed in 1994 with the National Natural Science Foundation of China, in 1995 with the Chinese Academy of Social Sciences and in 2007 with the Ministry of Sciences and Technologies. Before these official and institutional agreements, strong individual relations had been created between scientists, which were a necessary basis for further fruitful collaboration. The most important aspect is undoubtedly human mobility, and from both countries efforts were made with hosting of researchers in a framework of joint research projects or of joint research laboratories, with high priority given to involving young researchers.

Chemistry is oriented to finding new materials, new molecules and new processes, and generally trying to do it with economy wherever possible: reducing the energy necessary to design the processes, sparing the materials and decreasing the side products, reducing the size of the systems. This is illustrated via joint actions involving many academic centers all over China and in all regions of France, with exchanges taking place in both directions.

One of the very first domains of Sino-French collaboration focused on catalysis, more than 50 years ago between Lyon, Beijing and Dalian with the venue of Chinese scientists in France. This started officially with creation of a joint laboratory in 2000 in the field of petrochemistry. It is now continuing in the field of zeolites, including State Key Laboratories in Dalian, Changchun and Caen. This domain of research of new concepts, reactions, materials, bio-inspired molecules, surfactants, solvents, electro-catalysis and CO_2_ valorization is now the goal of the Shanghai International Laboratory involving East China Normal University, East China University of Science and Technology, Fudan University, Lyon University and Solvay, which we had the opportunity to initiate during the Shanghai Expo in 2010.

During my time as Director for Chemical Sciences in CNRS (2006–2011), I had the chance to promote and create several collaborative actions between France and China: in 2007, this was a joint laboratory in the field of Nano- and micro-electrochemistry between Xiamen University and ENS-Paris; in 2009, the creation of the international laboratory in Zhengzhou, dedicated to organophosphorus materials with the University of Rennes; and, more recently, in 2019, a network was initiated in the field of energy between Changchun and Grenoble.

I wish particularly to emphasize the creation in 2008 of the French–Chinese Network in Theoretical Chemistry initiated by Prof. Weihai Fang, which is still progressing between Beijing Normal University, Nanjing University, University of Science & Technology of China and more than 10 others in China and more than 20 laboratories all over France. This network is the source of many exchanges and common works between our two countries.

The French Chemical Society (SCF) and Chinese Chemical Society (CCS) are naturally supporting this international activity: our first official agreement SCF-CCS was signed in Paris in 2013 with Prof. Jiannian Yao, and this has been renewed regularly since then. Within this agreement we celebrate, every second year, lectureship awards with respective visits of Chinese chemists (Shigang Sun, He Tian and Zhigang Shuai have been the laureates) in France and French chemists (Pierre Dixneuf, Serge Cosnier and Michel Che) in China with a series of lectures in the countries.

The links between our two communities are stronger than ever and we take any opportunity to meet! It was the privilege of France to host the International Year of Chemistry in Paris in 2011 and the IUPAC General Assembly & Congress in 2019, under the Presidency of Prof. Qifeng Zhou, which celebrated the centenary of this organization.

